# Experimental medication treatment approaches for depression

**DOI:** 10.1038/tp.2017.33

**Published:** 2017-03-21

**Authors:** D F Ionescu, G I Papakostas

**Affiliations:** 1 Depression Clinical and Research Program, Department of Psychiatry, Massachusetts General Hospital, Boston, MA, USA; 2 Harvard Medical School, Boston, MA, USA; 3 Clinical Trials Network and Institute, Massachusetts General Hospital, Boston, MA, USA

## Abstract

Depression is one of the most common psychiatric conditions. Symptoms can lead to significant disability, which result in impairments in overall quality of life. Though there are many approved antidepressant treatments for depression—including selective serotonin reuptake inhibitors, tricyclic antidepressants and monoamine oxidase inhibitors—about a third of patients do not respond to these medications. Therefore, it is imperative for drug discovery to continue towards the development of novel and rapidly acting compounds, especially for patients with treatment-resistant depression. After a brief review of the efficacy of approved antidepressant therapies, we will discuss experimental medication treatments for depression. Specifically, we examine novel medications that are thought to primarily modulate the glutamatergic, cholinergic and opioid systems to achieve antidepressant efficacy. We also give examples of anti-inflammatories, neurokinin-1 modulators, vasopressin antagonists and neurogenesis enhancers that may have a therapeutic role in treatment-resistant depression. The current pipeline of antidepressant treatments is shifting towards medications with novel mechanisms, which may lead to important, life-changing discoveries for patients with severe disease.

## Introduction

Depression is one of the most devastating human conditions. In the Unites States alone, the economic burden attributed to major depressive disorder (MDD) increased by over 21% from 2005 to 2010; this cost is representative of the combination of direct effects, suicide-related expenditures and workplace costs.^1^ Several reasons may explain this sharp uptick in costs in a short period of time: an increase in both the population of the United States as well as in the prevalence of MDD; an increase in the costs of treatments; changes in the composition and quality of treatment services; and changes in the rates of employment and treatment-seeking behaviors.^1^ Without a doubt, the discovery of treatments to combat depression is essential.

Though there are several classes of approved antidepressant treatments for depression—including selective serotonin reuptake inhibitors, tricyclic antidepressants and monoamine oxidase inhibitors—about a third of patients do not respond to these medications.^2^ Even when patients do respond to available treatments, the effects often take weeks to months to meet their full potential. Furthermore, treatment-resistant depression (a form of depression that does not respond to one or more antidepressant treatments of adequate dose and duration) is severe and, in some cases, debilitating. For example, 17% of patients with treatment-resistant depression have a prior suicide attempt, underscoring the severe nature of this disease.^3^ In addition to lost productivity and poorer quality of life, in the United States alone, it is estimated that treatment-resistant depression costs nearly $50 billion annually.^3^ These reasons underscore the critical need for rapid and novel therapies for treatment-resistant depression.

After a brief review of the efficacy of standard antidepressants, we will discuss the status of experimental antidepressant therapeutics. The mechanism by which many of these novel antidepressants are thought to work represents a departure from traditional antidepressants (which may affect their antidepressant actions primarily through the modulation of monoaminergic neurotransmission). Specifically, new medications that modulate the glutamatergic system are at the forefront of antidepressant research and development.

### Antidepressant efficacy: a review of current standards and challenges

How effective are antidepressants in treating patients with MDD? Data from STAR*D—the largest comparison trial, to date, of antidepressant effectiveness among agents from different classes—support the use of antidepressants in patients with MDD, as there was a cumulative 67% response rate after all four steps of the study.^4^ Furthermore, in an analysis that was independent of the STAR*D data set, Gibbons *et al.*^5^ found an estimated response rate of 58.4% (versus 39.9% for placebo) to antidepressants. Indeed, antidepressants can be helpful to many patients.

However, antidepressant monotherapy alone may not always be enough to treat depression. Towards this end, augmentation strategies with other classes of medications can be a useful option.^2^ For example, a meta-analysis of antipsychotic efficacy in 16 trials (*n*=3480) found that adjunctive atypical antipsychotics significantly outperformed placebo as treatment strategies for depression response and remission (though their use is associated with an increased risk of discontinuation due to adverse events).^6^ Quetiapine and aripiprazole are approved by the US Food and Drug Administration (FDA) as adjunctive therapies for the treatment of depression, while olanzapine/fluoxetine combination therapy is approved for patients with treatment-resistant depression (as per the FDA definition ⩾2 failed treatments). Furthermore, ziprasidone was recently found to significantly outperform placebo in patients with depression (*n*=139) with regard to reductions in depression scores (as measured by the Hamilton Depression Rating Scale; HDRS).^7^ Similarly, 8-week adjunctive use of the atypical antipsychotic cariprazine 2–4.5 mg per day (*n*=276) was effective for treating depression in patients with refractory disease compared with cariprazine 1–2 mg per day (*n*=274) or placebo (*n*=269).^8^ Drug tolerability was favorable, adding to its potential promise for treatment-resistant depression.

Recently, however, psychiatric drug development has been plagued with failed trials.^9^ Though negative trials (in which it can be concluded with confidence that the experimental treatment is not superior in efficacy to placebo) are informative, failed trials (in which there is a high likelihood that the study, either by design or circumstance, was unable to detect a treatment difference from placebo) are largely inconclusive. Unfortunately, high placebo response rates in antidepressant medication studies may harm the outcome of clinical trials; failed results may prematurely end the development of otherwise potentially efficacious antidepressants. Data from one meta-analysis of 182 clinical trials (*n*=36 385) found that pooled response rates of antidepressants and placebo were 53.8% and 37.3%, respectively.^10^ Furthermore, showing superiority of drug versus placebo becomes difficult when placebo rates are ⩾30 and ⩾40% for monotherapy and adjunctive trials, respectively.^11^

Indeed, one major challenge in antidepressant medication research is to keep placebo response rates low. To address this issue, several solutions are proposed, including the sequential parallel comparison design^12^ and the use of external, blinded raters to assess clinical trial patients. Furthermore, unlocking the neurobiology of the placebo response may be an important advancement towards understanding the treatment of depression from a biological standpoint; this is an area of active investigation (ClinicalTrials.gov ID: NCT02562430).

Despite evidence that antidepressants and augmentation strategies work for 60–70% of people with major depression, our currently approved antidepressants do not work for many people. It is especially imperative that the development of new treatments continues for this group. Towards this end, new compounds are under active investigation. Here, we will review the current experimental treatments for depression, with a focus on compounds with novel mechanisms of action compared with currently approved antidepressant treatments.

## Experimental treatments for depression

### Glutamatergic agents

#### Ketamine: current state of research

Since its serendipitous discovery as a rapidly acting antidepressant over a decade and a half ago, ketamine research has increased steadily, as extensively reviewed in the literature.^13, 14^ Briefly, a single subanesthetic dose of intravenous ketamine consistently decreases symptoms of depression in patients with treatment-resistant depression in a rapid (within hours), robust (across many symptoms of depression) and relatively sustained (typically, 7–14 days) manner.^15, 16, 17^ Furthermore, ketamine significantly beat the active comparator midazolam in patients with severe treatment-resistant depression;^18^ this finding represents a major breakthrough (because of previous unblinding concerns with the use of a saline placebo). Ketamine is generally well tolerated and safe when given as a single treatment for research purposes,^19^ though questions remain about abuse potential, as well as transient psychotomimetic, sympathomimetic and dissociative side effects. Furthermore, the optimal dose of ketamine for depression remains unclear and may differ between patients. To address this, a multisite randomized, double-blind, placebo-controlled dose-finding trial is currently ongoing (ClinicalTrials.gov ID: NCT01920555).

Extending ketamine's antidepressant properties beyond 7–14 days has been somewhat of a ‘holy grail' in ketamine research. Towards this end, several groups have examined the use of multiple-dose regimens. Repeated doses (six infusions over the course of several weeks) have shown promise from an efficacy and safety standpoint,^16, 20^ though patients typically relapse within a few days to weeks after the final infusion—even when the dose is escalated from 0.5 mg kg^−1^ to 0.75 mg kg^−1^ hallway through the study, as evidenced by work from Cusin *et al.*^16^ Furthermore, Singh *et al.*^21^ recently evaluated the efficacy of two times versus three times a week intravenous ketamine in patients (*n*=67) with treatment-resistant depression in sustaining the initial antidepressant effects. Both doses were significantly superior to placebo in treating depression symptoms and the transient dissociative side effects attenuated with repeat dosing. There was no advantage of two times versus three times a week ketamine doses, as both maintained antidepressant efficacy over 15 days.

Indeed, there is great promise in ketamine's ability to decrease depression quickly in patients with severe treatment-resistant depression when given intravenously; however, the logistics of intravenous administration is a challenge (as this often requires hospital admission and consultation with an anesthesiologist). Therefore, several studies have examined alternate routes of ketamine administration for depression. Lapidus *et al.*^22^ compared a single intranasal administration of ketamine 50 mg versus saline placebo in patients (*n*=20) with treatment-resistant depression. At 24 h, ketamine significantly outperformed placebo with regard to decreases on depression rating scale scores; however, no significant difference between ketamine and placebo was appreciated by 72 h post-administration. Despite not being as sustained as intravenous ketamine, this study demonstrated that intranasal ketamine is well tolerated. Janssen, a subsidiary of Johnson & Johnson, is actively investigating the use of intranasal esketamine (the S-enantiomer of racemic ketamine) for the treatment of depression. Preliminary evidence for intranasal esketamine is promising; data from a Phase II study suggest that four administrations of intranasal esketamine over the course of 2 weeks significantly reduced depression scores (compared with placebo) in patients with severe treatment-resistant depression at three different doses (28, 56 and 84 mg).^23^ Further investigations into intranasal esketamine are underway (ClinicalTrials.gov IDs: NCT02417064, NCT02497287, NCT02418585). Because intranasal esketamine has received breakthrough therapy designation from the FDA, if ongoing studies are positive, approval will likely be fast-tracked.

Recently, Loo *et al.*^24^ assessed the assessed the feasibility, efficacy and safety of intravenous (*n*=4), intramuscular (*n*=5) and subcutaneous (*n*=6) routes for treating depression with ketamine (versus midazolam active comparator) in treatment-resistant depression patients. Doses were also titrated from 0.1 mg kg^−1^ up to 0.5 mg kg^−1^ in separate treatment sessions separated by at least 1 week. All three routes of administration resulted in comparable antidepressant effects, though the subcutaneous route was noted to have the fewest adverse effects. Though it is important to stress the small sample size of this study, these results provide preliminary evidence for (i) alternative routes of dosing other than intravenous methods and (ii) the use of lower doses than the standard 0.5 mg kg^−1^ for the treatment of depression.

As recently reviewed by Abdallah *et al.*,^25^ though ketamine's mechanism of antidepressant action remains unknown, its ability to manipulate the glutamatergic system may have an important role. Until recently, ketamine's *N*-methyl-d-aspartate (NMDA) antagonistic properties were considered the most likely mechanism towards the production of antidepressant effects. Specifically, ketamine blocks NMDA receptors. Among many actions, this leads to a release of the tonic gamma-aminobutyric acid inhibition from interneurons on the pre-synaptic glutamatergic neurons, thereby resulting in a glutamate ‘surge'. In turn, more glutamate is available in the synapse to activate prosynaptogenic α-amino-3-hydroxy-5-methyl-4-isoxazolepropionic acid (AMPA) receptors, thereby inducing synaptic plasticity via intracellular mechanisms of mTOR enhancement and increased brain derived neurotrophic factor production. Though these pathways are still considered a possible mechanism for ketamine's antidepressant effects, recently basic science work has revealed the utility of (2R,6R)-hydroxynorketamine—a metabolite of ketamine—via direct AMPA activation.^26^ Given that ketamine's plasma terminal half-life is 2.5 h,^27^ perhaps (2R,6R)-hydroxynorketamine helps to explain why ketamine's antidepressant properties typically extend through 1–2 weeks post infusion (and longer in some populations, such as those with anxious depression^28^ or a family history of alcoholism^29, 30^).

Because of ketamine's great promise as a rapidly acting antidepressant, many ketamine treatment clinics have opened (especially in the United States) over the past several years. However, its clinical use is still highly cautioned; there is no current long-term safety data to support ketamine's repeated use in depression beyond a few weeks. In addition, there are no standard operating procedures for how such a clinic should run, from the proper selection of patients to the dosing and route of administration. Until these data are available, potential clinic patients must be cautioned as to the unknown long-term side effects of this treatment.

To answer these questions, efforts and resources should focus on further research into ketamine's mechanism and safety. Towards this end, the intramural program at the National Institute of Mental Health has embarked on several mechanism of action studies (ClinicalTrials.gov IDs: NCT00088699 and NCT02122562), which combine clinical, neuropsychological, neuroimaging and sleep markers for ketamine's antidepressant effects. Other extramural studies, including one of our own studies (which aims to investigate psychophysiological and cognitive biomarkers of ketamine's antidepressant effects (ClinicalTrials.gov ID: NCT02669043)) are ongoing. The hope is that from these mechanism studies, drug development may progress towards new compounds with novel mechanisms of action (similar to ketamine). In addition to its promise as a model for the discovery of rapidly acting antidepressants, studies into ketamine's antidepressant mechanism may also provide a pathway to understand the pathophysiology of treatment-resistant depression in patients, thereby contributing to the elucidation of mental illness.

### Other glutamatergic modulators

Despite the excitement that ketamine has provided towards the discovery of rapidly acting antidepressants with novel mechanisms of action, many unsuccessful efforts have resulted from investigations into other compounds with glutamatergic modulating activity. However, a few compounds remain promising. Here, we will review both the recent successes and disappointments within this category.

#### Riluzole

Though approved for use in amyotrophic lateral sclerosis, riluzole has also been studied for treatment-resistant depression. Its mechanisms are thought to involve the inhibition of glutamate release, NMDA-receptor antagonism and AMPA enhancement.^31^ One open-labeled study of riluzole monotherapy for patients with treatment-resistant depression (*n*=19) found that riluzole use resulted in significant improvements in depression symptoms.^32^ Another open-label study found that riluzole, when used as an augmentation agent to ongoing antidepressant treatments, resulted in significant improvements of symptoms of anxiety and depression.^33^ However, data from many other studies argue against riluzole's usefulness in treatment-resistant depression. Several studies have examined riluzole's theoretical potential to extend the antidepressant effects of ketamine after a single infusion with no avail.^34, 35^ Furthermore, riluzole does not appear to decrease symptoms of depression in patients that were unresponsive to ketamine.^36^ In the largest study of riluzole augmentation to date, 104 patients with treatment-resistant depression were randomized to either riluzole (50 mg b.i.d.) or placebo in a 56-day study that utilized sequential parallel comparison design.^37^ Even with an overall low placebo response rate (<30%), riluzole did not outperform placebo on mean change in Montgomery Asberg Depression Rating Scale (MADRS) scores (*P*=0.83). Despite some initial excitement, riluzole's antidepressant properties do not appear to be as convincing as those found with ketamine.

#### Lanicemine (AZD 6765)

Lanicemine, developed by AstraZeneca, is a moderate-affinity, low-trapping NMDA-receptor antagonist that is administered intravenously; based on mechanism alone, it was hypothesized to possess rapidly acting antidepressant properties. An initial study in medication-free patients with treatment-resistant depression (*n*=22) suggested that a single infusion of lanicemine 150 mg significantly reduced depression scores (compared with placebo) within 80 min of administration; interestingly, psychotomimetic and dissociative side effects did not differ from placebo, suggestive of a potential advantage over ketamine.^38^ Unfortunately, this improvement in depression scores only remained significant through 110 min.

Lanicemine's antidepressant efficacy was further investigated in two subsequent trials.^39^ In the first, patients with MDD (*n*=34) were randomized to receive a single infusion of lanicemine 100 mg versus placebo. Despite significant improvements in depression scores at several secondary time points for lanicemine compared with placebo, the primary outcome measure—change in MADRS scores at 24 h—did not differ significantly from placebo. Of note, the placebo response was large, with an average decrease in scores on placebo of more than 14 points. In the next study, currently medicated patients with MDD (*n*=124) were randomized to repeat infusions of adjunctive lanicemine 100 mg, 150 mg or placebo; infusions took place three times a week for 3 weeks and symptoms were observed for five additional weeks. Both doses of lanicemine significantly decreased depression scores from baseline to week 3 compared with placebo; for patients in the 100 mg dose group, this significant improvement lasted up through the 5 weeks of observation. Again, lanicemine demonstrated no significant side effects (psychotomimetic or dissociative). However, these significant findings did not replicate in a larger multisite trial, as the placebo response rate was 39% at primary end point.^40^ Nonetheless, data from the lanicemine studies have shown that an NMDA-receptor antagonist may potentially have antidepressant properties without inducing unwanted psychotomimetic or dissociative side effects.

#### Memantine

Memantine is an oral medication approved and marketed for Alzheimer's disease. One of its properties—low-to-moderate-affinity NMDA-receptor blockade—made it an attractive candidate as an antidepressant. However, trials of memantine for depression were unsuccessful. In one trial (an 8-week, double-blind, placebo-controlled study) of patients with major depressive disorder (*n*=32), memantine monotherapy (5–20 mg per day) did not beat placebo in reducing depression scores.^41^ Similarly, results from another trial did not find a significant difference between placebo and memantine augmentation (20 mg per day).^42^ In summary, little evidence exists to support the efficacy of memantine for MDD.

#### Traxoprodil (CP-101,606)

As opposed to the previously discussed non-competitive NMDA-receptor antagonists of ketamine, riluzole, lanicemine and memantine, traxoprodil is an intravenous NMDA-receptor antagonist specific to the NR2B subunit. Preskorn *et al.*^43^ administered adjunctive traxoprodil or placebo to paroxetine-resistant patients with depression (*n*=30). Traxoprodil produced a significantly greater decrease versus placebo at the prespecified outcome measure of change in MADRS score from baseline to day 5 at the 10% level of significance; the response rate with traxoprodil was 60% versus 20% on placebo. Furthermore, response was maintained for 1 week in 78% of initial responders. These data provide evidence for the need for further research into the antidepressant properties of selective NR2B blockade.

#### CERC-301 (MK-0657)

Similar to traxoprodil, MK-0657 is also an NR2B selective NMDA-receptor antagonist. Originally developed as a Merck compound (MK-0657), and later, further developed by Cerecor (CERC-301), this drug was studied as an oral monotherapy antidepressant treatment in unmedicated patients with treatment-resistant depression.^44^ In this randomized, double-blind, placebo-controlled crossover study, no significant differences (*P*=0.27) in antidepressant efficacy were found between MK-0657 and placebo on the primary outcome measure (as measured by MADRS); however, secondary outcome measures were promising. Specifically, MK-0657 outperformed placebo for decreases on the HDRS (*P*=0.001) and Beck Depression Inventory (*P*=0.01). In addition, there were no dissociative or other serious adverse events. Unfortunately, only five patients (out of the 21 planned) completed both periods of the crossover portion of the study (in part due to recruitment challenges and discontinuation of the compound by the manufacturer). Nonetheless, CERC-301 currently remains in Phase II development; a current trial for adjunctive CERC-301 was completed with results pending (ClinicalTrials.gov identifier: NCT01941043).

### Metabotropic glutamate receptor modulators

Straying from drugs with mechanisms involving the ionotropic NMDA, AMPA and kainate glutamatergic receptors, several compounds have been developed as antagonists of the metabotropic receptors of the glutamatergic system. Specifically, Roche (Basel, Switzerland) has developed both basimglurant (RG7090 or RO4917523)—a mGluR5 receptor antagonist, and decoglurant (RG1578 or RO4995819)—a mGluR2/3 antagonist. An initial study of adjunctive decoglurant therapy was completed, with results pending (ClinicalTrials.gov ID: NCT01457677). Results from Phase II testing of basimglurant have, however, been published. Specifically, in one randomized, double-blind study, patients with MDD (*n*=333) who were partial or non-responders to antidepressant therapy received once daily adjunctive basimglurant 0.5 or 1.5 mg, or placebo, for 6 weeks.^45, 46^ Though well tolerated, there was no statistically significant difference between basimglurant versus placebo for change in the clinician-rated MADRS from baseline to end point—the study's primary outcome measure (effect size=0.16, *P*=0.42). Of note, there was a sizeable response rate in the control group (more than 14 points on the MADRS). Indeed, secondary end points—particularly patient-rated measures—found a significant antidepressant effect for basimglurant 1.5 mg compared with placebo. For example, larger improvements were seen for basimglurant versus placebo on the patient-rated MADRS (*P*=0.04). Though there is little evidence to support efficacy based on the primary outcome measure, mixed results from the secondary end points of basimglurant 1.5 mg may point towards the utility for the development of future studies on mGluRs as potential targets for antidepressant therapies.

#### Rapastinel (GLYX-13)

Rapastinel (formally known as GLYX-13) is an intravenously administered functional partial agonist at the glycine-binding site on the NMDA receptor. In preclinical rat studies, rapastinel had rapid antidepressant efficacy without the same side effect burden (for example, psychotomimetic effects) of ketamine.^47^ Similarly, human studies have been promising. Specifically, Preskorn *et al.*^48^ completed a randomized, double-blind trial in which patients (*n*=116) with treatment-resistant depression (unresponsive to ⩾1 monoaminergic treatment in the current episode) received either rapastinel (at doses 1, 5, 10 or 30 mg kg^−1^) or placebo. Rapastinel's antidepressant effects were noted within 2 h in the 5 mg kg^−1^ and 10 mg kg^−1^ group as measured by the HDRS (the primary efficacy outcome); furthermore, the antidepressant effects continued to separate from placebo through day 7.^48^ Long-term safety and efficacy research is ongoing (ClinicalTrials.gov ID: NCT02192099). Rapastinel has received the FDA's breakthrough therapy designation.

#### AVP-786 (dextromethorphan/quinidine)

AVP-786 is an experimental compound developed by Avanir (Aliso Viejo, CA, USA). A combination of deuterium-modified dextromethorphan hydrobromide and ultra-low dose quinidine sulfate, AVP-786 is similar to Nudexta, a medication approved to treat pseudobulbar affect. (Of note, a trial of Nuedexta for treatment-resistant depression (NCT01882829) was recently completed; results are pending). Mechanistically, dextromethorphan is an uncompetitive NMDA-receptor antagonist, as well as a sigma-1 receptor agonist; these properties theoretically have antidepressant effects. Low-dose quinidine (a CYP2D6 enzyme inhibitor) works to increase the bioavailability of dextromethorphan by inhibiting its breakdown. In addition, the deuterium incorporation into dextromethorphan contributes to its bioavailability by strengthening chemical bonds within the molecule, making it less susceptible to metabolic breakdown. Together, this combination may serve to bypass metabolic breakdown of the molecule, allowing for increased NMDA-receptor antagonism by dextromethorphan in the brain. A clinical trial of AVP-786 as adjunctive therapy in MDD was recently completed with results pending (ClinicalTrials.gov ID: NCT02153502).

#### AXS-05 (dextromethorphan/bupropion)

In addition to the NMDA-receptor blocking properties of dextromethorphan, AXS-05 contains bupropion (a norepinephrine and dopamine reuptake inhibitor that is currently approved for the treatment of depression), which may also work to increase the bioavailability of dextromethorphan in the brain. Axsome (New York, NY, USA) is currently conducting Phase III trials (ClinicalTrials.gov ID: NCT02741791). Specifically, patients will have a 6-week lead-in period with open-label bupropion, followed by a 6-week, double-blind treatment period to compare the efficacy of AXS-05 augmentation to bupropion versus bupropion monotherapy in patients with treatment-resistant depression (defined as failure of one to two antidepressant treatments in the current episode and a treatment failure to the lead-in trial of bupropion). Because quinidine can cause cardiac toxicity when used excessively, AXS-05 may offer a theoretical advantage over AVP-786 in patients at a risk of overdose or with cardiac conduction concerns.

### Opioid modulators

Dysregulation of the endogenous opioid system may contribute to the pathophysiology of MDD.^49^ Thus, opioid modulation in the brain may have profound antidepressant treatment effects. Here, we review several compounds that may lead to novel treatments for depression via this system.

#### AZD2327

Based on preclinical data, Richards *et al.*^50^ recently investigated AZD2327—a selective delta-opioid receptor agonist—for the treatment of anxious depression (a particularly difficult-to-treat depression subtype^51^). In the double-blind, placebo-controlled pilot study in humans (*n*=22), patients received 4 weeks of either AZD2327 (3 mg b.i.d.) or placebo. Although seven (54%) patients responded to AZD2327 (compared with three (33%) patients who responded to placebo), there were no significant differences between the two arms on measures of depression or anxiety. Interestingly, levels of one of the major metabolites of AZD2327 (AZ12311418) were significantly higher (*P*=0.03) in patients that had an anxiolytic response compared with non-responders. No current trials of AZD2327 are active at this time, though this study may provide some preliminary evidence for the potential anxiolytic effects of delta-opioid receptor agonism.

#### ALKS 5461 (buprenorphine/samidorphan)

The combination of buprenorphine (a partial agonist at the mu- and antagonist kappa-opioid receptors FDA approved for the treatment of opioid addiction) and samidorphan (μ-opioid receptor antagonist)—known as ALKS 5461—leads to functional kappa-opioid antagonism, a mechanism that theoretically should result in a consistent antidepressant effect. Towards this end, ALKS 5461 was recently studied as adjunctive therapy for patient with treatment-resistant depression in a large multicenter, randomized, double-blind, placebo-controlled, two-stage sequential parallel comparison design study.^52^ Patients were randomized to 2 mg/2 mg of buprenorphine/samidorphan, 8 mg/8 mg of buprenorphine/samidorphan or placebo. After 4 weeks, there were significantly greater antidepressant effects in the group of patients that received 2 mg of each drug compared with placebo (*P*<0.01 on MADRS). Furthermore, ALKS 5461 demonstrated good tolerability, with no concerns for opioid withdrawal or tolerance at the conclusion of the study. Two placebo-controlled MDD studies were recently completed for ALKS 5461 (ClinicalTrials.gov IDs: NCT02158533, NCT02158546). In addition, there is an ongoing efficacy trial that is actively recruiting (ClinicalTrials.gov ID: NCT02218008), as well as an open-label long-term (52-week) safety and tolerability trial (ClinicalTrials.gov ID: NCT02141399).

### Cholinergic modulators

#### Scopolamine

Hypercholinergic states in the brain may be a cause of depression.^53^ Therefore, anticholinergic compounds may have a role in the treatment of depression. Scopolamine, derived from the plant *Brugmansia*, is an antimuscarinic medication that acts specifically at the M1 receptor. One study investigated intravenous repeat infusions of scopolamine (4 mcg kg^−1^) versus placebo.^54^ Specifically, patients with unipolar or bipolar depression (*n*=19) were randomized to receive a sequence of scopolamine/placebo or placebo/scopolamine; each series consisted of three placebo sessions and three scopolamine sessions at 3–5 days apart. Compared with placebo, scopolamine significantly reduced symptoms of depression and anxiety after only a few days after the initial intravenous scopolamine infusion. These data were later replicated in a patient with major depressive disorder (*n*=22 analyzed).^55^

#### CP-601,927

Developed by Pfizer, CP-601,927 is a partial agonist at the nicotinic acetylcholine receptor. Fava *et al.*^56^ recently conducted a randomized, double-blind, placebo-controlled trial to evaluate the antidepressant efficacy of augmentation with CP-601,927 (1–2 mg b.i.d.) compared with placebo in patients with treatment-resistant depression (refractory to selective serotonin reuptake inhibitors). Unfortunately, there were no overall statistical differences between changes in depression ratings from baseline to week 14 in the drug group compared with placebo. However, *post hoc* analyses revealed that elevated leptin levels at baseline had a significant effect on treatment outcome. Specifically, *P*-values for the effect of treatment on changes in depression scores were 0.0055 for patients above the median for leptin levels. Despite the overall negative results, the data point toward a potential use of leptin levels as a biomarker of patient subtypes for predicting treatment outcomes.

### Anti-inflammatory agents

#### Sirukamab

Sirukamab is a monoclonal antibody that also has anti-inflammatory properties against the pro-inflammatory cytokine interleukin-6. Originally designed for the treatment of rheumatoid arthritis, sirukamab may also have antidepressant properties, as increased interleukin-6 (a marker of inflammation) may be implicated in the pathophysiology of depression.^57^ Unpublished data from a *post hoc* analysis demonstrated decreases in symptoms of depression, anhedonia and fatigue at week 12 in patients undergoing treatment with sirukamab for rheumatoid arthritis.^58^ Currently, Janssen is conducting a clinical trial to evaluate the antidepressant efficacy of adjunctive subcutaneous sirukumab compared with placebo in patients with depression (ClinicalTrials.gov ID: NCT02473289).

### Neurokinin-1 antagonists

#### Aprepitant, casopitant and orvepitant (GW823296)

Substance P, which is a neurotransmitter and a neuromodulator, has pro-inflammatory properties and acts on neurokinin-1 (NK-1) receptors. Initial excitement about the theoretical antidepressant properties of an NK-1 antagonist were dampened when one such compound—aprepitant—failed to separate from placebo in five separate randomized clinical trials.^59^ Subsequently, the argument was proposed that NK-1 antagonists required maximal central nervous system (CNS) occupancy (close to 100%) in order to be efficacious in MDD.^60^ Two separate randomized, placebo-controlled studies of the NK-1 antagonist casopitant for MDD have been published to date.^61^ Doses used in these trials had previously^62^ been shown to yield nearly 100% NK-1-receptor occupancy in the CNS by positron emission tomography scans. In the first trial, 80 mg (*P*=0.023), but not 30 mg (*P*=0.07) of casopitant demonstrated greater efficacy on the HDRS than placebo (reference placebo response rate of, approximately, eight points). In the second trial, both flexible dose casopitant (80–120 mg) and paroxetine failed to separate from placebo (reference placebo response rate of, approximately, 12 points).

More recently, orvepitant—an NK-1 antagonist—was proposed for further study, due to its full and long-lasting saturation of central NK-1 receptors that separated it from prior similar compounds. Like casopitant, doses shown to have near-full CNS NK-1 receptor occupancy by positron emission tomography were selected.^60^ In two clinical trials, patients with depression were randomized to receive orvepitant 30 mg daily, 60 mg daily or placebo. Both orvepitant 30 mg and 60 mg daily demonstrated significant antidepressant efficacy over placebo in one study (*n*=328) at 6 weeks (reference placebo response rate of, approximately, nine HDRS points). In a separate study with a similar placebo response rate, 30 mg daily of orvepitant demonstrated superior antidepressant efficacy compared with placebo at weeks 1 and 2 (but not 4 and 6), while the 60 mg group demonstrated greater antidepressant efficacy than placebo at all time points except week 6.^60^ Given these encouraging results for orvepitant and casopitant, further development of NK-1 antagonists with doses aiming for near-100% CNS NK-1 receptor occupancy should be pursued.

### Vasopressin antagonists

#### Nelivaptan (SSR149415)

Vasopressin works through pituitary and central vasopressin receptors to control the hypothalamic–pituitary–adrenal axis, as well as several emotional processes;^63^ therefore, the modulation of vasopressin receptors may be a mechanism for antidepressant drug development. Developed by Sanofi-Aventis Laboratories (Toulouse, France), SSR149415 is an oral nonpeptide vasopressin V(1b) receptor antagonist. Griebel *et al*.^64^ reported data from three randomized, double-blind, 8-week trials of SSR149415 for MDD. In a paroxetine-controlled MDD trial evaluating two doses of SSR149415 (100 mg b.i.d., 250 mg b.i.d.), only the paroxetine treatment arm separated from placebo (*P*=0.006). Of note, the placebo response in this likely uninformative study was approximately 13 points on the HDRS. Interestingly, the results of an escitalopram-controlled trial did demonstrate statistically greater efficacy for the 250 mg b.i.d. treatment group (*P*=0.024), with a reference placebo response in that study of approximately nine HDRS points. Although no statistically significant difference in efficacy between SSR149415 100 mg b.i.d. or 250 mg b.i.d. was noted in a third MDD study, mean changes in HDRS scores in placebo treatment groups were not reported to help assess the extent to which the studies were informative. Though the results are mixed, further studies are necessary to determine the antidepressant efficacy and safety of this or similar compounds.

### Neurogenesis enhancers

#### NSI-189

Evidence exists to suggest that decreased hippocampal volume may be involved in the pathophysiology of depression. Conversely, reversal of this deficit may be an important target for the development of novel antidepressants.^65^ Towards this end, NSI-189 is a benzylpiperizine-aminiopyridine compound developed by Neuralstem (Germantown, MD, USA) with neurogenic properties (though its exact mechanism remains unknown). Recently, a Phase 1b study was completed in patients with depression (*n*=24).^66^ In this double-blind, placebo-controlled study, patients were randomized to one of three arms; one group received 40 mg daily of NSI-189 (*n*=6) or placebo (*n*=2); one group received 40 mg b.i.d. (*n*=6) or placebo (*n*=2); and the final group received 40 mg t.i.d. (*n*=6) or placebo (*n*=2) for 28 days. NSI-189 was well tolerated at all doses, and reductions in depression measures showed promise. Despite the low number of patients per arm and the exploratory nature of this study, NSI-189 has potential as a novel antidepressant; further research in MDD is ongoing (ClinicalTrials.gov ID: NCT02695472).

## Conclusion

Many patients with depression do not respond to our currently available antidepressant treatments, all of which primarily exert their immediate mechanism of action through monoaminergic modulation. Therefore, it is imperative that depression research moves towards compounds that target non-monoaminergic molecular structures. Towards this end, there are several exciting paths of development underway. Ketamine has been, by far, the most studied of all the novel compounds within the past two decades. This has propelled a wide range of research into other agents with glutamatergic modulatory mechanisms. Given the success and replication of ketamine trials, evidence is building for its potential use as a rapidly acting antidepressant for treatment-resistant depression, as indicated by the number of ongoing studies. Furthermore, ketamines may provide a model for understanding the mechanisms behind rapidly acting antidepressants, which may lead to the discovery of novel compounds to treat depression.

One major problem with clinical trials in depression is the issue of failed or uninformative trials (as opposed to the generally more informative negative studies). As the data suggest, several experimental compounds do fail to separate from placebo in terms of efficacy. However, many studies lack the use of a standard therapy study arm. Through the addition of both a placebo arm and a standard therapy comparator group, results of studies can be more definitively characterized as negative versus failed trials when the experimental compound does not outperform the comparators. In other instances, the high placebo response rates in depression clinical trials and a low ‘signal to noise' ratio have likely resulted in the abandonment of several potentially promising compounds. This problem also highlights the importance of selecting appropriate study subjects for clinical trials. For example, confirming illness severity, level of treatment resistance and diagnosis through structured interviews by external and experienced psychologists and psychiatrists is emerging as a standard design feature in the field for quality assurance.^67^

From a research perspective, the placebo effect itself remains a topic under active investigation. Recent data suggest that the μ-opioid system is likely implicated in the formation of placebo antidepressant effects in depressed patients.^68^ Further studies on the mechanisms of placebo response are ongoing (ClinicalTrials.gov ID: NCT02562430, NCT01787240). Indeed, the discovery of the neurobiological underpinnings of placebo responses may lead to other novel compounds for treating depression—and beyond.

Despite many past disappointments, the pipeline for novel medications for the treatment of depression is growing. Most of the excitement is currently aimed towards compounds that modulate glutamatergic neurotransmission. Certainly, great caution is taken along with this excitement, as many compounds that were initially promising failed to stand the test of clinical trials.^69^ However, for our field to move forward, it is important that we remain optimistic. It is only since the 1950s that antidepressants (as we know them today) came to the clinical research arena.^70^ Perseverance and a deeper understanding of how to distinguish negative from uninformative studies will be critical for the continued discovery of novel antidepressants.
